# Systematic review protocol of interventions to improve the psychological well-being of general practitioners

**DOI:** 10.1186/s13643-015-0098-z

**Published:** 2015-09-22

**Authors:** Marylou Murray, Lois Murray, Michael Donnelly

**Affiliations:** School of Medicine, Dentistry and Biomedical Sciences, Centre for Public Health, Queen’s University Belfast (QUB), Institute of Clinical Sciences, Royal Victoria Hospital, Block B, Belfast, BT 12 6BA UK; King’s College Hospital, Denmark Hill, London, SE5 9RS UK; School of Medicine, Dentistry and Biomedical Sciences, Centre for Public Health and UKCRC Centre of Excellence for Public Health Northern Ireland, Queen’s University Belfast (QUB), Institute of Clinical Sciences, Royal Victoria Hospital, Block B, Belfast, BT 12 6BA UK

**Keywords:** General practitioner, Well-being, Mental health promotion, Prevention

## Abstract

**Background:**

The challenges and complexities faced by general practitioners are increasing, and there are concerns about their well-being. Consequently, attention has been directed towards developing and evaluating interventions and strategies to improve general practitioner well-being and their capacity to cope with workplace challenges.

**Methods/design:**

This systematic review aims to evaluate research evidence regarding the effectiveness of interventions designed to improve general practitioner well-being. Eligible studies will include programmes developed to improve psychological well-being that have assessed outcomes using validated tools pertaining to well-being and related outcomes. Only programmes that have been evaluated using controlled study designs will be reviewed. An appropriately developed search strategy will be applied to six electronic databases: the Cochrane Database of Systematic Reviews, MEDLINE, Embase, CINAHL, PsycINFO and Web of Science. Studies will be screened in two stages by two independent reviewers. A third reviewer will arbitrate when required. Pre-specified inclusion and exclusion criteria will be assessed during a pilot phase early on in the review process. The Cochrane data extraction form will be adapted and applied to each eligible study by two independent reviewers, and each study will be appraised critically using standardised checklists from the Cochrane Handbook. Methodological quality will be taken into account in the analysis of the data and the synthesis of results. A narrative synthesis will be undertaken if data is unsuited to a meta-analysis.

**Discussion:**

The systematic review will be reported according to the Preferred Reporting Items for Systematic Reviews and Meta-analyses guidance. This will be the first systematic review on this topic, and the evidence synthesis will aid decision-making by general practitioners, policy makers and planners regarding ways in which to improve GP well-being. Findings will be disseminated at general practitioner meetings, conferences and in professional and peer-reviewed journals.

**Systematic review registration:**

PROSPERO CRD42015017899

**Electronic supplementary material:**

The online version of this article (doi:10.1186/s13643-015-0098-z) contains supplementary material, which is available to authorized users.

## Background

A recent comparative study of health systems in 11 countries including the USA, Australia and the UK ranked the UK National Health Service (NHS) system first in many parameters and indicated that the degree to which a system was based on a primary care model appeared to be related positively to the delivery of high-quality, efficient and cost-effective care—even when the size of a country’s healthcare budget was taken into account [1]. An ageing primary care population with increasing co-morbidities requires the holistic, generalist management offered by general practitioners (GPs) especially in the context of increasing sub-specialisation within secondary care and rapid advances in treatment. The primary care model provides a practice and delivery forum for the co-ordination of services, interpretation of investigations and management of medication. However, there is a pressing need to address current workforce and workload stressors within primary care, and dedicated effort is required to prevent mental ill health among GPs including burnout, work-related stress, addiction, depression and suicide.

A concentrated focus on improving GP well-being is likely to improve retention problems as well as impacting positively on the recruitment of new and returning GPs. Workload stressors include increases in the volume and complexity (e.g. increasing multi-morbidity) of clinical work with increasing administrative and bureaucratic demands, decreased funding and changing political agendas [2, 3]. A UK Department of Health review in 2014 confirmed that the GP workforce is under pressure and predicted a major demand-supply imbalance within 5 years. Recruitment of GPs is becoming more difficult [4]. Currently, general practice is viewed as an increasingly ‘pathogenic environment’ [5–7]. Its previous appeal as a flexible, family-friendly career choice is waning with GP daily workloads typically involving 60 patient contacts and many GPs reporting that they spend their evenings and weekends completing paperwork [8]. Compounding recruitment issues, there are increasing concerns about the retention of GPs in the context of a tide of increasing regulation, complaints, patient demands and loss of autonomy. The privilege of caring for patients, which has been the bedrock of satisfaction [9], is under constant scrutiny by regulators and the media. Yet, despite reports of maleficence (e.g. the Mid-Staffs Hospital Inquiry [10]) and increases in complaints to the GMC [11], recent data show that patients are satisfied and happy with their GP [12]. General practice appears to be producing high-quality patient and performance outcomes but at considerable personal cost to practitioners [13, 14].

Whilst constructs such as satisfaction, well-being and performance tend to be measured using psychometric instruments, it is important to bear in mind the reductionist nature of such tools and the results of research that use them. Wallace et al. conceptualise wellness as a quality indicator and cite factors such as fatigue due to workload, vicarious trauma, maladaptive coping mechanisms and stigmatisation as determinants of physician ill health. The impact of work organisational variables upon doctors’ health has personal and professional implications especially regarding patient safety. Wallace et al.’s recommendation to measure physician wellness raises debate about the quality and appropriateness of tools, the meaning of ‘wellness’ and the provision and delivery of services for physicians who are identified as having less ‘wellness’ and for ‘secondary victims’ (healthcare workers traumatised following an adverse event) [15, 16].

Recent advances in well-being research may provide a solution, at least in part, to the challenges and complexities described above. This paper presents a protocol for the first systematic review of interventions that have been designed to improve GP well-being.

A scoping exercise of the various conceptualisations of well-being informed the protocol. Whilst there is a lack of agreement about the definition of well-being, a synthesis of recent work by key researchers in the field of well-being [17–20] appears to indicate that it is a multidimensional construct that comprises the core dimensions of (i) positive affect, (ii) personal relationships and social engagement and (iii) a life view that is meaningful and optimistic. The key elements or dimensions of well-being that emerged from the scoping exercise guided the writing of the review protocol (see Table 1).

**Table 1:** Well-being constructs across key theories

Element of well-being	Diener 2010 [17]	Huppert 2013 [18]	Seligman 2012 [19]	Tennant 2007 [20]
Self-acceptance	Yes	Yes		Yes
Competence	Yes	Yes		Yes
Relationships	Yes	Yes	Yes	Yes
Presence of positive affect	Yes	Yes	Yes	Yes
Meaning	Yes	Yes	Yes	Yes
Optimism	Yes	Yes		Yes
Engagement	Yes	Yes	Yes	
Contribution to well-being of others	Yes			Yes
Purpose in life	Yes			
Absence of negative affect	Yes			
Life satisfaction	Yes			
Emotional stability		Yes		
Resilience		Yes		
Vitality/energy		Yes		Yes
Accomplishment			Yes	
Cheerfulness				Yes
Relaxation				Yes
Clear thinking				Yes
Personal development				Yes
Autonomy				Yes

These well-being theories incorporate the concepts of ‘happiness’ and ‘flourishing’. The latter is considered to be an optimal state of well-being and as constituting the presence of mental health whilst ‘languishing’ represents an absence of mental health [21]. An examination of available relevant scales in relation to these theoretical perspectives is instructive regarding the operationalisation of the construct of well-being. For example, Diener [17] based his new scale for measuring *flourishing* on earlier work by Ryff [22, 23], Ryan and Deci [24], Putnam [25] and Helliwell et al. [26], and it incorporates a theory of psychological capital that includes ‘life flow’, interest and engagement [27], optimism [28] and Seligman’s earlier conceptualisation of happiness [29]. The flourishing scale developed by Huppert and So [18] situates well-being as sitting on a continuum that comprises the opposite constructs of the symptoms of common mental disorders defined in the International Classification of Diseases 10 [30] and the Diagnostic and Statistical Manual of Mental Disorder 4 [31]. The scale overlaps considerably with Diener’s operationalisation of well-being, and in turn, these conceptualizations are similar to Seligman’s 2012 [19] theory of well-being and to the conceptualisation that underpins the Warwick-Edinburgh Scale of Well-being [20]. Whilst there is considerable common ground between these views, each one has a unique aspect which this review will incorporate in order to conduct a thorough review of efforts to improve GP well-being. Furthermore, in recognition of the varied and inconsistent ways in which the term well-being and related terms are used and with the aim of undertaking an international and comprehensive review of well-being interventions for GPs and primary care physicians, the review will consider, at least initially, interventions and programmes that are referred to as addressing psychological well-being, mental health and mental well-being and associated terms.

## Methods

The systematic review including its methodology will be reported according to the Preferred Reporting Items for Systematic Reviews and Meta-analyses (PRISMA) guidance [32]. (See Additional file 1 for PRISMA P Checklist.)

An initial scoping exercise indicated that there were a disproportionate number of studies relating to interventions delivered by GPs relative to GPs being the recipients of an intervention. Therefore, as indicated above, the review will adopt a broad, inclusive approach to the selection of studies. Eligibility criteria will ensure the capture of GPs as the population of interest and their well-being as the target or outcome focus. Interventions designed primarily to improve patient care (including interventions designed to improve GP knowledge and skills in patient care) will be excluded.

PICOS details are outlined in Table 2.

**Table 2:** A summary of the PICOS elements that comprise the systematic review

Population	Intervention	Comparison	Outcome	Study design
Primary care doctors working alone, in group practices and healthcare teams in any country	Any (psychosocial) intervention or combination of interventions developed to improve any element or combination of elements of well-being.	No intervention or usual support and care	Primary: all well-being outcomes related to any of the 20 aspects of well-being identified in Table 1 or combinations thereof including improvement in mental illness, measures of stress, anxiety, depression, addiction and burnout	Randomised controlled trials including cluster randomised trials
				Non-randomised controlled trials
				Controlled before-and-after trials
				Interrupted time series trials
			Secondary: physiological outcome measures of improvement in well-being, e.g. cortisol.	

### Population

General practitioners or synonyms, e.g. family practitioners, primary care physicians, family practice and general practice, comprise the population of this systematic review.

### Intervention

Interventions that address any element or combination of the elements of well-being noted above will be considered eligible for inclusion. The conceptual nature of the operational definition of well-being in each study (and, therefore, whether or not it should be included in the review) will be assessed by Marylou Murray (MM) and Lois Murray (LM). A consensus will be derived via discussion and by comparing each independent assessment with the elements of well-being listed in Table 1. A third independent reviewer Michael Donnelly (MD) will be consulted when there is reviewer disagreement. Interventions targeted at primary and secondary prevention levels are eligible for inclusion. Primary interventions include, for example, programmes that address job design and the management of work in order to minimise harm and efforts to promote protective factors at individual, group and organisational levels. Secondary level interventions include those which promote and facilitate early help-seeking behaviours and those addressing increased mental health awareness including stigmatisation [33].

### Comparators

No intervention or usual care will serve as the comparator for this systematic review.

### Outcome measures

Primary outcome measures will include mental well-being, mental health and mental ill-health measures using validated tools. Measures of mental illness will be considered in recognition of their use in studies purporting to measure well-being. Also, interventions which reduce mental health problems or mental illness symptoms may advance a person along the well-being continuum from languishing towards flourishing—though it is accepted that languishing is not synonymous with mental illness. Possible examples are reported in Table 3.

**Table 3:** Examples of measures used to assess well-being

Outcome measured	Instrument
Mental well-being	Warwick-Edinburgh Mental Wellbeing Scale [20]
	Diener’s Flourishing Scale [17]
	General Health Questionnaire (GHQ) [34]
	Physician Job Satisfaction Scale [35]
Burnout	Maslach’s Burnout Inventory [36]
Psychological distress	Perceived Stress Scale (PSS) [37]
	Distress sub-scale of Symptom Checklist Rev (SCL-R) [38]
Anxiety	Depression Anxiety Distress Scale [39]
	Brief Symptom Inventory [40]
	Anxiety sub-scale of SCL-R
Depression	Beck’s Depression Inventory [41]
	Brief Symptom Inventory
	Depression sub-scale of SCL-R
	Center for Epidemiology Studies-Depression (CES-D) scale [42]
	DASS
Empathy	Interpersonal Reactivity Index (IRI) [43]
	Jefferson’s Scale Physician Empathy [44]
Mindfulness	Freiberg Mindfulness Inventory (FMI) [45]
Quality of life	WHOQOL [46]

### Study design

The study design will comprise randomised controlled trials including cluster randomised trials, non-randomised controlled trials, controlled before-and-after trials and interrupted time series trials. Uncontrolled studies will be excluded.

Studies of the well-being of doctors who are not working in primary care, e.g. hospital physicians and occupational health doctors, will be excluded. Studies designed to improve knowledge and clinical skill for patient management will also be excluded.

Interventions designed to be delivered at the tertiary level to GPs recovering from mental ill health such as rehabilitation, return to work programmes and treatments for severe mental illness will be excluded.

#### Language

An English language restriction will be applied due to the heterogeneity in terminology and inconsistency in well-being constructs across languages.

#### Publication type

Studies published in peer-reviewed journals will be considered in this systematic review.

#### Identifying the research base

A search strategy will be developed with the assistance of a specialist subject librarian. Synonyms for the population will be identified and included in the search strategy. Informed by a preceding scoping review, the review team will search six databases: the Cochrane Database of Systematic Reviews, MEDLINE, Embase, PsycINFO, CINAHL and Web of Science including the Social Science Citation Index. These databases will be searched from inception until a date which will be cited. References in eligible titles will be hand searched independently by MM and LM.

Study selection will be a two stage process.

Stage 1 Initial screening of all titles and abstracts will be performed independently by MM and LM. MD will provide additional quality control by screening a randomly selected 10 % of titles.

Titles will be assessed against the aforementioned inclusion and exclusion criteria. Excluded titles will be categorised into two groups:

- ○ 1. Clearly not relevant—will be recorded as ‘not relevant’
- ○ 2. Do not meet all inclusion criteria—reasons will be recorded for this group

Stage 2 MM and LM will assess the full texts of studies appearing to meet inclusion criteria in order to ascertain eligibility for inclusion.

#### Pilot

A pilot of study selection will be undertaken during which MM and LM will apply the inclusion criteria to a 10 % sample of titles identified by the search. This pilot will enable refinement of inclusion criteria and facilitate inter-assessor consistency and reliability. MM and LM will discuss disagreements and then discuss further with MD when it proves difficult to arrive at a consensus.

#### Duplication

Titles will be managed within Refworks. Initial de-duplication will be performed by MM using the ‘exact duplicates’ facility within this reference management system. Further de-duplication will be undertaken by MM during the screening of titles.

Decisions on all titles will be recorded.

#### Flow chart

Study selection will be illustrated using the PRISMA format.

A sample data extraction form will be designed which will include the following:

- Identification features—number, author, title and country of origin
- Study characteristics—aims/objectives, design, recruitment and unit of allocation
- Participant characteristics—age and gender
- Intervention—setting and description of intervention including control, duration, theoretical basis and format
- Outcome/results—for each outcome, the definition, measurement tool, unit of measurement and length of follow-up

Regarding the intervention and control group in each study, we will collect data on the number of participants included in the analysis, number of withdrawals, exclusions and participants lost to follow-up and summary of outcomes—dichotomous and continuous, type of analysis used and results (OR, RR, mean differences and confidence limits). This extraction form will be piloted and modified as required.

Each eligible study will be appraised critically for key methodological aspects such as the appropriateness of study design, risk of bias, choice of outcome measures, statistical issues, quality of reporting, quality of intervention and generalisability. The critical appraisal will be facilitated via the use of standardised checklists as recommended in the Cochrane Handbook 2011. The potential impact of methodological quality will be taken into account in the synthesis of the data and the results.

MM and LM will assess independently each eligible study for risk of bias using the risk of bias tool (Cochrane Handbook 2011) including the generation and concealment of allocation, blinding, how missing or incomplete data were handled and potential for selective outcome reporting. The quality of the data according to the level of risk of bias may lead to a differentiated analysis. Relative risk and 95 % confidence intervals (CIs) will be calculated for dichotomous outcomes whilst mean differences and 95 % CIs will be calculated for continuous outcomes. Standardised mean differences (SMDs) will be used to group continuous outcomes that were derived using different measures. We will extract and assess data at baseline and all subsequent follow-up measurement points. Regarding the missing data, we will enquire from the relevant research team the possibility of obtaining the data. We will attempt to calculate any missing SMDs for continuous measures from the reported statistics (e.g. CIs and SEs) in the relevant paper. The statistical heterogeneity of studies will be assessed using RevMan and calculating the *χ*^2^ test and *I*^2^ statistic. We will sum the studies using a random effect model if there is substantial heterogeneity or we will use a fixed effect model when heterogeneity is low. One or more sensitivity analyses will assess the impact of omitting or including given (sets of) studies. A funnel plot will plot trial effect against standard error.

A clear tabulated description of studies will initiate the data synthesis stage. Key features will include study type, intervention, outcome and outcome measure. We will conduct a meta-analysis (according to the Cochrane Handbook) if the studies and their data are of good quality and ‘fit’ together reasonably well. The results will be synthesised in a narrative format if a meta-analysis is not possible or sensible to undertake. The narrative synthesis will follow an iterative process in terms of stages of theory development, preliminary synthesis, exploration of relationships and assessment of the robustness of synthesis. According to the results of our initial scoping exercise, we plan to group and analyse the measurement of outcome data in terms of baseline and 6-month or later time points. As mentioned above, a differentiated or sensitivity analysis will assess the impact on review results of including then excluding studies of variable methodological rigour and risk bias. Decisions regarding synthesis will be guided by best practice and discussed by the review team.

## Discussion

The systematic review will be reported according to the PRISMA guidelines. This will be the first systematic review of interventions designed to improve the well-being of general practitioners. The current context of workplace stressors in primary care and the pivotal role played by a GP in healthcare delivery to ageing multi-morbid populations highlights the urgent need to prevent work-related ill health and improve GP well-being.

## Additional file

Additional file 1:
**PRISMA-P 2015 checklist.** The list contains recommended items to include in a systematic review protocol.

## Abbreviations

GPs: general practitioners
LM: Lois Murray
MD: Michael Donnelly
MM: Marylou Murray
NHS: National Health Service
