# Supplementary material for: Systematic review protocol of interventions to improve the psychological well-being of general practitioners
Source: Syst Rev. 2015 Sep 22;4:117. doi: 10.1186/s13643-015-0098-z (PMC4580348; doi:10.1186/s13643-015-0098-z)
Supplement: Additional file 1: — PRISMA-P 2015 checklist. The list contains recommended items to include in a systematic review protocol. [file 13643_2015_98_MOESM1_ESM.pdf]

PRISMA-P 2015 checklist: recommended items to include in a systematic review protocol[a]

Section/topic	Item #	Checklist item
**ADMINISTRATIVE INFORMATION**		
**Title Systematic review protocol of interventions to improve the psychological well-being of general practitioners**		
**Identification**	1a	Identified as protocol in title
**Update**	1b	This is the first document and not an update
**Registration**	2	PROSPERO reference CRD42015017899
**Authors**		
**Contact**	3a	Marylou Murray, School of Medicine, Dentistry and Biomedical Sciences, Centre for Public Health, Queen's University Belfast (QUB), Institute of Clinical Sciences, Block B<br>Royal Victoria Hospital<br>Belfast BT12 6BA<br>mmurray881@qub.ac.uk.<br>Lois Murray, King's College Hospital, London<br>Loismurray1@nhs.net<br>Michael Donnelly, School of Medicine, Dentistry and Biomedical Science, Centre for Public Health, QUB & UKCRC Centre of Excellence for Public Health, QUB. michael.donnelly@qub.ac.uk
**Contributions**	3b	All authors have made substantive contributions to the conception and design of the protocol and have given approval for this final version. MM and MD were involved in drafting and revising the document critically for important intellectual content. MM is the guarantor.
**Amendments**	4	This is the original protocol. Important protocol amendments shall be documented in full and submitted to the registering body in compliance with PROSPERO registration
**Support**		
**Sources**	5a	MM is in receipt of a PhD studentship
**Sponsor**	5b	Department for Education and Learning, Northern Ireland

PRISMA-P 2015 checklist: recommended items to include in a systematic review protocol[a]

Section/topic	Item #	Checklist item
**Role of sponsor/funder**	5c	The funder did not have any direct role in the development of this protocol
**INTRODUCTION**		
**Rationale**	6	An aging population with increasing co-morbidities requires the holistic, generalist management offered by GPs especially in the context of increasing sub-specialisation within secondary care and rapid advances in treatment. The primary care model provides a practice and delivery forum for the co-ordination of services, interpretation of investigations and management of medication. However, there is a pressing need to address current workforce and work-load stressors within primary care and dedicated effort is required to prevent mental ill-health among GPs including burnout, work-related stress, addiction, depression and suicide. General practice appears to be producing high quality patient and performance outcomes but at considerable personal cost to practitioners. Recent advances in well-being research may provide a solution, at least in part, to the challenges and complexities described above.
**Objectives**	7	This systematic review aims to evaluate research evidence for the effectiveness of interventions designed to improve GP well-being. An initial scoping exercise indicated that there were a disproportionate number of studies relating to GPs delivering interventions relative to GPs as the recipients of interventions. The review will adopt a broad, inclusive approach to the selection of studies. Eligibility criteria will ensure the capture of GPs as the population of interest and their well-being as the target or outcome focus. Interventions designed primarily to improve patient care (including interventions designed to improve GP knowledge and skills in patient care) will be excluded<br>Population: General practitioners or synonyms e.g. family practitioners, primary care physicians, family practice, general practice.<br>Intervention: In recognition of the heterogeneous nature of the term ‘well-being’, interventions that address any element or combination of the elements of well-being identified in synthesis of published constructs and definitions of well-being (presented in tabular format) will be considered eligible for inclusion. Marylou Murray (MM) and Lois Murray (LM) will compare their independent assessments of the eligibility of interventions against the twenty

PRISMA-P 2015 checklist: recommended items to include in a systematic review protocol[a]

Section/topic	Item #	Checklist item
		elements (in Table X).<br>Outcomes - primary: Any one or more of the 20 aspects of well-being identified in the synthesis of well-being constructs as well as improvement in measures of stress, anxiety, depression, addiction and burnout; secondary: physiological measures of improvement in well-being e.g. cortisol
**METHODS**		
**Eligibility criteria**	8	**Outcome measures**: Primary outcomes that are measured using validated tools. Inclusion of measures such as depression acknowledges evidence pointing to their use in studies of well-being. Interventions which decrease, for example, anxiety and depression may advance a participant along the well-being continuum from languishing towards flourishing (though it is recognised that languishing is not synonymous with mental ill-health). **Exclusion criteria:** Doctors who are not working in primary care e.g. hospital physicians and occupational health doctors; studies designed to improve GP clinical knowledge and skills regarding patient management; studies without a control group or condition; and interventions delivered to GPs at tertiary level (eg rehabilitation and return-to-work programmes and treatments for severe mental illness).<br>**Eligible study designs:** Randomized controlled trials including cluster randomized trials; non-randomized controlled trials; controlled before-and-after trials; and interrupted time series trials<br>**Language**: An English language restriction has been applied due to the heterogeneity in terminology and inconsistency in well-being constructs across languages.<br>**Publication type:** Studies published in peer-reviewed journals.<br>**Identifying the research base:** A search strategy will be developed with the assistance of a specialist subject librarian. Synonyms describing the population of interest will be identified and included in the search strategy in order to capture internationally recognised terminology.
**Information sources**	9	Informed by a preceding scoping review, the review team will search six databases: Cochrane Database of Systematic Reviews, Medline, Embase, PsychINFO, CINAHL and Web of Science including Social Science Citation Index. These databases will be

PRISMA-P 2015 checklist: recommended items to include in a systematic review protocol[a]

Section/topic	Item #	Checklist item
		searched from inception until a date which will be cited. References in eligible titles will be hand searched independently by MM and LM.<br><br>Search Strategy:
Search strategy	10	

#	Searches
1	"positive psychology intervention*".mp. [mp=title, abstract, original title, name of substance word, subject heading word, keyword heading word, protocol supplementary concept word, rare disease supplementary concept word, unique identifier]
2	mindfulness.mp. [mp=title, abstract, original title, name of substance word, subject heading word, keyword heading word, protocol supplementary concept word, rare disease supplementary concept word, unique identifier]
3	"life review".mp. [mp=title, abstract, original title, name of substance word, subject heading word, keyword heading word, protocol supplementary concept word, rare disease supplementary concept word, unique identifier]
4	("positive writing" or gratitude or "hope therapy" or "well-being therapy" or "wellbeing therapy").mp. [mp=title, abstract, original title, name of substance word, subject heading word, keyword heading word, protocol supplementary concept word, rare disease supplementary concept word, unique identifier]
5	(cognitive adj3 therap*).mp. [mp=title, abstract, original title, name of substance word, subject heading word, keyword heading word, protocol supplementary concept word, rare disease supplementary concept word, unique identifier]
6	("forgiveness therapy" or "positive reminiscence").mp. [mp=title, abstract, original title, name of substance word, subject heading word, keyword heading word, protocol supplementary concept word, rare disease supplementary concept word, unique identifier]
7	"Fordyce's happiness".mp. [mp=title, abstract, original title, name of substance word, subject heading word, keyword heading word, protocol supplementary concept word, rare disease supplementary concept word, unique identifier]
8	(Fordyce adj5 happiness).mp. [mp=title, abstract, original title, name of substance word, subject heading word, keyword heading word, protocol supplementary concept word, rare disease supplementary concept word, unique identifier]
9	("cultivating sacred moment*" or "sacred moment*").mp.

PRISMA-P 2015 checklist: recommended items to include in a systematic review protocol[a]

Section/topic	Item #	Checklist item
		[mp=title, abstract, original title, name of substance word, subject heading word, keyword heading word, protocol supplementary concept word, rare disease supplementary concept word, unique identifier]
	10	"life coach*".mp. [mp=title, abstract, original title, name of substance word, subject heading word, keyword heading word, protocol supplementary concept word, rare disease supplementary concept word, unique identifier]
	11	(discuss* adj5 belief*).mp. [mp=title, abstract, original title, name of substance word, subject heading word, keyword heading word, protocol supplementary concept word, rare disease supplementary concept word, unique identifier]
	12	"rehearsal of positive statement*".mp. [mp=title, abstract, original title, name of substance word, subject heading word, keyword heading word, protocol supplementary concept word, rare disease supplementary concept word, unique identifier]
	13	(rehearsal adj5 positive).mp. [mp=title, abstract, original title, name of substance word, subject heading word, keyword heading word, protocol supplementary concept word, rare disease supplementary concept word, unique identifier]
	14	kindness*.mp. [mp=title, abstract, original title, name of substance word, subject heading word, keyword heading word, protocol supplementary concept word, rare disease supplementary concept word, unique identifier]
	15	(optimism or optimistic).mp. [mp=title, abstract, original title, name of substance word, subject heading word, keyword heading word, protocol supplementary concept word, rare disease supplementary concept word, unique identifier]
	16	(cognitive adj3 restructur*).mp. [mp=title, abstract, original title, name of substance word, subject heading word, keyword heading word, protocol supplementary concept word, rare disease supplementary concept word, unique identifier]
	17	(emotional adj5 regulat*).mp. [mp=title, abstract, original title, name of substance word, subject heading word, keyword heading word, protocol supplementary concept word, rare disease supplementary concept word, unique identifier]
	18	"emotional learning".mp. [mp=title, abstract, original title, name of substance word, subject heading word, keyword heading word, protocol supplementary concept word, rare disease supplementary concept word, unique identifier]
	19	"compassion meditation".mp. [mp=title, abstract, original title,

PRISMA-P 2015 checklist: recommended items to include in a systematic review protocol[a]

Section/topic	Item #	Checklist item
		name of substance word, subject heading word, keyword heading word, protocol supplementary concept word, rare disease supplementary concept word, unique identifier]
	20	(compassion adj5 meditation).mp. [mp=title, abstract, original title, name of substance word, subject heading word, keyword heading word, protocol supplementary concept word, rare disease supplementary concept word, unique identifier]
	21	(secular adj5 meditation*).mp. [mp=title, abstract, original title, name of substance word, subject heading word, keyword heading word, protocol supplementary concept word, rare disease supplementary concept word, unique identifier]
	22	(signature adj5 strength*).mp. [mp=title, abstract, original title, name of substance word, subject heading word, keyword heading word, protocol supplementary concept word, rare disease supplementary concept word, unique identifier]
	23	("wellbeing intervention*" or "well-being intervention*").mp. [mp=title, abstract, original title, name of substance word, subject heading word, keyword heading word, protocol supplementary concept word, rare disease supplementary concept word, unique identifier]
	24	(brief adj5 intervention*).mp. [mp=title, abstract, original title, name of substance word, subject heading word, keyword heading word, protocol supplementary concept word, rare disease supplementary concept word, unique identifier]
	25	(wellbeing or (well adj being) or hedoni* or euda#monic* or eudemonic* or happ* or thriv* or flourish* or languish* or (life adj5 satisf*) or autonom* or (environment* adj5 master*) or (personal adj5 growth) or (relation* adj5 other*) or (positive adj5 affect*) or (negative adj5 affect*) or selfacceptance or (self adj acceptance) or sad*).mp. [mp=title, abstract, original title, name of substance word, subject heading word, keyword heading word, protocol supplementary concept word, rare disease supplementary concept word, unique identifier]
	26	Depression/
	27	exp Anxiety/
	28	stress.mp.
	29	Psychopathology/
	30	psychomorbid*.mp. [mp=title, abstract, original title, name of substance word, subject heading word, keyword heading word, protocol supplementary concept word, rare disease supplementary concept word, unique identifier]

PRISMA-P 2015 checklist: recommended items to include in a systematic review protocol[a]

Section/topic	Item #	Checklist item
		31 Mental Health/
		32 ("mental health" adj5 improv*).mp. [mp=title, abstract, original title, name of substance word, subject heading word, keyword heading word, protocol supplementary concept word, rare disease supplementary concept word, unique identifier]
		33 25 or 26 or 27 or 28 or 29 or 30 or 31 or 32
		34 interven*.mp. [mp=title, abstract, original title, name of substance word, subject heading word, keyword heading word, protocol supplementary concept word, rare disease supplementary concept word, unique identifier]
		35 33 and 34
		36 1 or 2 or 3 or 4 or 5 or 6 or 7 or 8 or 9 or 10 or 11 or 12 or 13 or 14 or 15 or 16 or 17 or 18 or 19 or 20 or 21 or 22 or 23 or 24
		37 35 or 36
		38 General Practitioners/
		39 physicians, family/ or physicians, primary care/
		40 (GP* or "G.P*").mp. [mp=title, abstract, original title, name of substance word, subject heading word, keyword heading word, protocol supplementary concept word, rare disease supplementary concept word, unique identifier]
		41 "family doctor*".mp. [mp=title, abstract, original title, name of substance word, subject heading word, keyword heading word, protocol supplementary concept word, rare disease supplementary concept word, unique identifier]
		42 38 or 39 or 40 or 41
		43 stress, psychological/ or burnout, professional/
		44 34 and 43
		45 36 or 37 or 44
		46 42 and 45
		47 limit 46 to (English language and humans)
**Study records**		
**Data management**	11a	We will use Refworks for data management (as it is the system supported by Queen's University Belfast).
**Selection process**	11b	***Stage 1*** Screening of every title and abstract will be performed independently by MM and LM. MD will provide additional quality control by screening 10% of titles at this stage.

PRISMA-P 2015 checklist: recommended items to include in a systematic review protocol[a]

Section/topic	Item #	Checklist item
		Titles will be assessed against the aforementioned inclusion and exclusion criteria. Excluded titles will be categorized into two groups:<br>o 1. Clearly not relevant – will be recorded as 'not relevant'<br>o 2. Do not meet all inclusion criteria – reasons will be recorded for this group<br>***Stage 2*** MM and LM will assess the full text of each paper that appears to meet inclusion criteria in order to confirm or reject their eligibility status.<br>*Pilot* A pilot of study selection will be undertaken during which MM and LM will apply the inclusion criteria to a 10% sample of titles identified by the search. This pilot will enable refinement of inclusion criteria and facilitate inter-assessor consistency and reliability. MM and LM will discuss disagreements and then discuss further with MD when it proves difficult to arrive at a consensus<br>*Duplication* Titles will be managed within Refworks. Initial de-duplication will be performed by MM using the 'exact duplicates' facility within this reference management system. Further de-duplication will be undertaken by MM during screening of titles.<br>*Decisions on all titles will be recorded*<br>*Flow chart* Study selection will be illustrated using the PRISMA format.
**Data collection process**	11c	A sample data extraction form will be designed which will include<br>• Identification features; number, author, title, country of origin<br>• Study characteristics; aims/objectives, design, recruitment, unit of allocation<br>• Participant characteristics; age, gender<br>• Intervention; setting, description of intervention including control, duration, theoretical basis, format.<br>• Outcome/results; for each outcome, the definition, measurement tool, unit of measurement, length of follow-up.<br>Regarding the intervention and control group in each study, we will collect data on the number of participants included in the analysis, number of withdrawals, exclusions and participants lost to follow-up, summary of outcomes – dichotomous and continuous, type of analysis used, results (OR, RR, mean differences and confidence limits). This extraction form will be piloted and modified as

PRISMA-P 2015 checklist: recommended items to include in a systematic review protocol[a]

Section/topic	Item #	Checklist item
		required.
**Data items**	12	See above
**Outcomes and prioritization**	13	Outcomes will include any well-being outcomes related to any of the 20 aspects of well-being identified in well-being construct synthesis or combinations thereof including improvement in psychopathology measures of stress, anxiety, depression, addiction and burnout. Priority will be given to these. Secondary outcomes which are physiological measurements of well-being such as cortisol having lower priority.
**Risk of bias in individual studies**	14	MM and LM will assess independently each eligible study for risk of bias using the risk of bias tool (Cochrane Handbook, 2011) including the generation and concealment of allocation, blinding, how missing or incomplete data were handled and potential for selective outcome reporting.
**Data**		
**Synthesis**	15a	The quality of the data according to the level of risk of bias may lead to a differentiated analysis. Decisions on synthesis will be informed by an experienced reviewer following detailed descriptive tabulation of data extracted. A clear tabulated description of studies will initiate the data synthesis stage. Key features will include study type, intervention, outcome and outcome measure. We will conduct a meta-analysis (according to the Cochrane Handbook) if the studies and their data are of good quality and ‘fit’ together reasonably well.
	15b	Relative risk and 95% Confidence Intervals (CI) will be calculated for dichotomous outcomes whilst mean differences and 95% CIs will be calculated for continuous outcomes. Standardised mean differences will be used to group continuous outcomes that were derived using different measures. We will extract and assess data at baseline and all subsequent follow-up measurement points. Regarding missing data, we will enquire from the relevant research team the possibility of obtaining the data. We will attempt to calculate any missing SDs for continuous measures from the reported statistics (eg CIs and SEs) in the relevant paper. The statistical heterogeneity of studies will be assessed using RevMan

PRISMA-P 2015 checklist: recommended items to include in a systematic review protocol[a]

**Section/topic**	**Item #**	**Checklist item**
		and calculating the χ^2 test and I^2 statistic. We will sum studies using a random effects model if there is substantial heterogeneity and we will use a fixed effects model when heterogeneity is low. One or more sensitivity analyses will assess the impact of omitting or including given (sets of) studies. A funnel plot will plot trial effect against standard error.
	15c	A differentiated or sensitivity analysis will assess the impact on review results of including then excluding studies of variable methodological rigour and risk bias.
	15d	The results will be synthesised in a narrative format if a meta-analysis is not possible or sensible to undertake. The narrative synthesis will be an iterative process between stages of theory development, preliminary synthesis, exploration of relationships and assessment of the robustness of synthesis.
**Meta-bias(es)**	16	.One or more sensitivity analyses will assess the impact of omitting or including given (sets of) studies. A funnel plot will plot trial effect against standard error.
**Confidence in cumulative evidence**	17	The strength of the body of evidence will be assessed using methodology from the 2011 Cochrane Handbook including the Cochrane 'Risk of bias' assessment tool and the Cochrane Effective Practice and Organisation of Care (EPOCH) group.

PRISMA-P Preferred Reporting Items for Systematic review and Meta-Analysis Protocols.

The copyright for PRISMA-P (including checklist) is held by the PRISMA-P Group and is distributed under a Creative Commons Attribution License 4.0.

Moher *et al.*

Moher *et al. Systematic Reviews* 2015 **4**:1 doi:10.1186/2046-4053-4-1

OPEN DATA
